# Lineage Tracing of Lamellocytes Demonstrates *Drosophila* Macrophage Plasticity

**DOI:** 10.1371/journal.pone.0014051

**Published:** 2010-11-19

**Authors:** Martin Stofanko, So Yeon Kwon, Paul Badenhorst

**Affiliations:** Institute of Biomedical Research, University of Birmingham, Edgbaston, England; Institut Pasteur, France

## Abstract

Leukocyte-like cells called hemocytes have key functions in *Drosophila* innate immunity. Three hemocyte types occur: plasmatocytes, crystal cells, and lamellocytes. In the absence of qimmune challenge, plasmatocytes are the predominant hemocyte type detected, while crystal cells and lamellocytes are rare. However, upon infestation by parasitic wasps, or in melanotic mutant strains, large numbers of lamellocytes differentiate and encapsulate material recognized as “non-self”. Current models speculate that lamellocytes, plasmatocytes and crystal cells are distinct lineages that arise from a common prohemocyte progenitor. We show here that over-expression of the CoREST-interacting transcription factor Chn in plasmatocytes induces lamellocyte differentiation, both in circulation and in lymph glands. Lamellocyte increases are accompanied by the extinction of plasmatocyte markers suggesting that plasmatocytes are transformed into lamellocytes. Consistent with this, timed induction of Chn over-expression induces rapid lamellocyte differentiation within 18 hours. We detect double-positive intermediates between plasmatocytes and lamellocytes, and show that isolated plasmatocytes can be triggered to differentiate into lamellocytes in vitro, either in response to Chn over-expression, or following activation of the JAK/STAT pathway. Finally, we have marked plasmatocytes and show by lineage tracing that these differentiate into lamellocytes in response to the *Drosophila* parasite model *Leptopilina boulardi*. Taken together, our data suggest that lamellocytes arise from plasmatocytes and that plasmatocytes may be inherently plastic, possessing the ability to differentiate further into lamellocytes upon appropriate challenge.

## Introduction

Leukocytes play a critical role in the innate immune responses of humans and invertebrates. Like their human counterparts, *Drosophila* leukocyte-like cells (termed hemocytes) neutralize invading pathogens through phagocytosis, encapsulation and melanization. Three hemocyte cell types have been shown to occur (reviewed in [1]–[3]). Of these, the most abundant is the plasmatocyte, which accounts for 95% of circulating hemocytes. Plasmatocytes are professional macrophages that are capable of removing foreign material and apoptotic cells by phagocytosis [4]. Less abundant are crystal cells, which comprise approximately 5% of hemocytes. Crystal cells are responsible for melanin synthesis during pathogen encapsulation and are required for wound healing and coagulation [5]. The final hemocyte type is a large flattened cell, the lamellocyte. These are responsible for encapsulating material recognized as “non-self”. Lamellocytes are seldom detected in larval hemolymph in the absence of immune challenge. However, large numbers differentiate either upon infestation by parasitic wasps [6], [7], or in a number of melanotic mutant strains [8], [9].

Two waves of hematopoiesis occur. First, during embryonic development hemocytes arise from the head mesoderm [10], [11] and subsequently populate the entire embryo. Following hatching of the larva, these hemocytes of embryonic origin persist and replicate in the larval hemolymph, while a second wave of hematopoiesis occurs in the larval lymph gland [11]–[13]. Hemocytes generated in the lymph gland are usually liberated at metamorphosis and contribute to the pupal and adult hemocyte populations [11]. As *Drosophila* exhibit an open circulatory system, hemocytes circulate in hemolymph (blood) that freely bathes all organs. In larva, movement of the hemolymph is mediated by contractions of the dorsal vessel and by peristaltic movements of the body. In a mature third instar larva approximately 5000 hemocytes occur, of which two thirds freely circulate in the hemocoel, while the remainder attach to the inner surface of the integument [12]. Both plasmatocytes and crystal cells are detected in these distinct sessile compartments [12], [14]. The function of these sessile compartments is unclear, although it has been proposed that they provide a progenitor pool for lamellocytes [15].

Current models for the origin of the three hemocyte types propose that plasmatocytes, crystal cells and lamellocytes represent distinct lineages that arise separately from a common stem cell or prohemocyte [1]–[3], [12]. The transcriptional hierarchy controlling plasmatocyte and crystal cell development is well characterized. In embryos, initial hemocyte determination requires the GATA factor Serpent (Srp) [16]. Subsequent differentiation into plasmatocytes is regulated by the transcription factors Glial cells missing (Gcm) and Gcm2 [17]. In contrast, differentiation into crystal cells is triggered by expression of the AML-1/Runx1 homologue Lozenge [18]. Lamellocyte differentiation can be triggered by activation of a number of signalling pathways including the JAK/STAT [9], [19], Toll [20] and JNK pathways [21]. Recently it has been suggested that the Friend of GATA protein U-Shaped (Ush) may be the target of these pathways during lamellocyte differentiation [22], [23]. Significantly, these authors suggest that lamellocytes arise from plasmatocytes, indicating that a reassessment of lamellocyte origin is required.

In the course of a misexpression screen to identify novel regulators of hemocyte development [14], we uncovered the transcriptional repressor *CG11798* (also known as *charlatan* (*chn*) or *taco*). Chn was first isolated in a genetic screen for mutations affecting embryonic peripheral nervous system (PNS) development [24] and was subsequently shown to be an important regulator of *achaete/scute* gene expression [25]. Chn is a putative transcription factor that contains 5 zinc fingers and shares similarity with the human neuronal restrictive silencing factor/repressor element RE-1 silencing transcription factor (NRSF/REST) [26]. Importantly, Chn interacts with the *Drosophila* homologue of the NRSF/REST co-repressor, CoREST [26]. CoREST forms the core of a transcription repressor complex that includes CtBP, HDAC-1/HDAC-2 and LSD1, and exhibits histone deacetylase and histone demethylase activities [27]–[29]. Through its associated repressor complexes NRSF/REST binding is speculated to erase histone marks of active transcription (H3K4me2 and H3K9Ac), establish the repressive histone mark (H3K9me2), and repress transcription [27]. In this report we show that Chn over-expression is able to trigger lamellocyte differentiation. Interestingly, Chn over-expression appears capable of transforming plasmatocytes into lamellocytes. Our results suggest an alternative pathway for the origin of lamellocytes.

## Results

### Chn overexpression increases lamellocyte number

**Figure 1:**
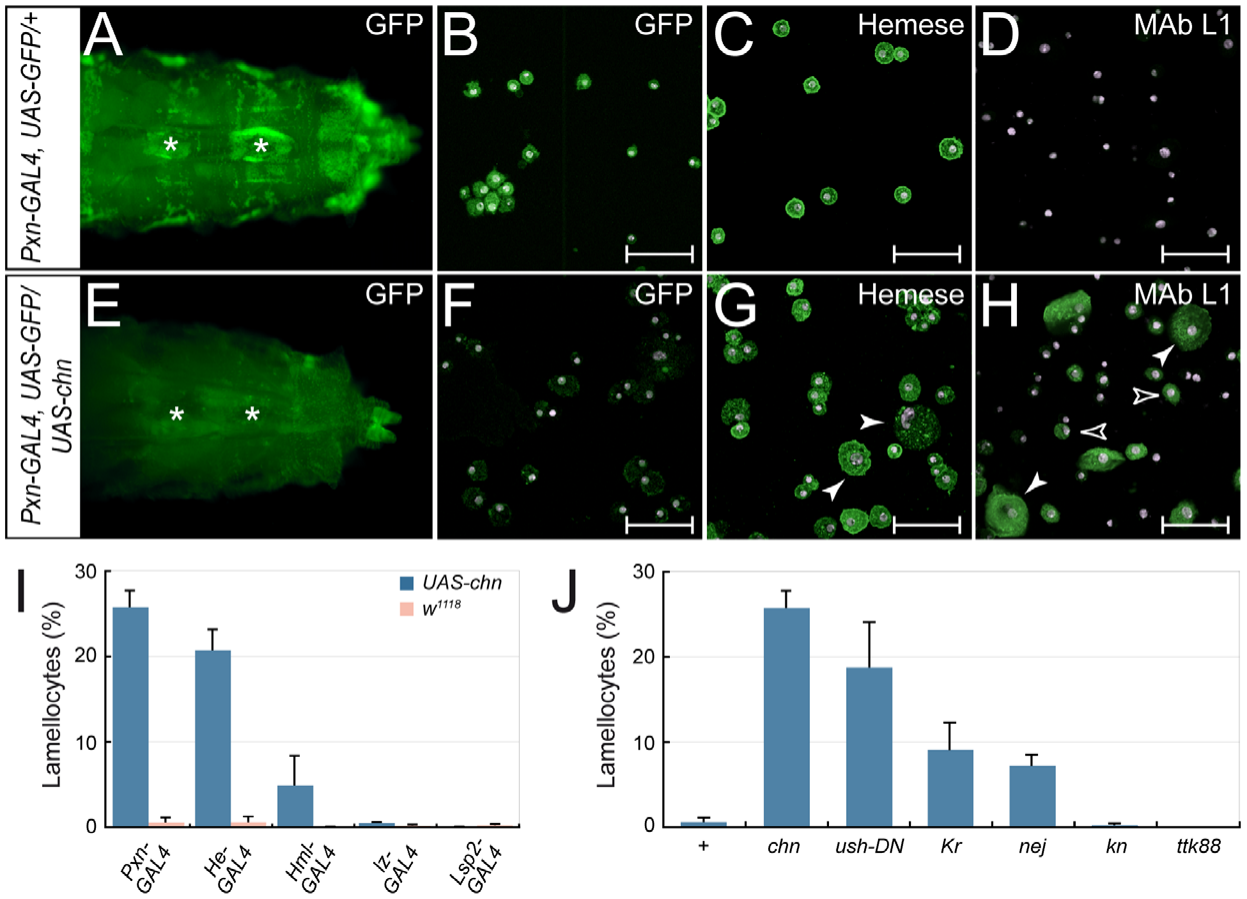
Chn over-expression increases lamellocyte number. Chn overexpression decreases (**A**,**E**) sessile (asterisk) and (**B**,**F**) circulating plasmatocyte numbers. (**C**, **G**) Anti-Hemese staining detects hemocytes after Chn expression although hemocyte size is increased (arrowheads) (**D**,**H**) MAb L1 staining confirms that Chn expression increases lamellocyte number. Circulating hemocytes were isolated from (**B–D**) *Pxn-GAL4*, *UAS-GFP x w^1118^* and (**F–H**) *Pxn-GAL4*, *UAS-GFP x UAS-chn* third instar larvae. Antibody staining is shown in green, DAPI-stained nuclei in purple. Scalebars denote 50 µm. (**I**) Lamellocyte frequency after Chn expression driven by different GAL4 lines. Control driver cross with *w^1118^* provides a reference. (**J**) Comparison of lamellocyte differentiation induced by expression of other transcription factors with *Pxn-GAL4*. Each data point in (**I**,**J**) is the mean +/− s.d. of at least 5 assays.

During a screen to identify new regulators of hemocyte development [14] we uncovered the putative transcriptional repressor CG11798 (also known as Charlatan (Chn) or Taco [24]–[26]). To investigate further the effect of Chn on hemocyte development we overexpressed Chn in larval plasmatocytes and crystal cells using the *Peroxidasin-GAL4* (*Pxn-GAL4*) driver. The *Pxn-GAL4* driver line also contains a *UAS-GFP* reporter that enables plasmatocytes and crystal cells to be visualized live in the semitransparent larvae. *Pxn-GFP* expressing sessile plasmatocytes (Fig. 1A) and circulating plasmatocytes (Fig. 1B) could be detected in the *Pxn-GAL4* driver control but were rarely observed in *Pxn-GAL4 x UAS-chn* larvae (Fig. 1E,F). Interestingly, loss of Pxn-GFP expression was not due to the absence of hemocytes as staining using the pan-hemocytic marker anti-Hemese [30] revealed that hemocytes were still present in Chn over-expressing larvae. Rather, hemocytes observed after Chn over-expression were larger than typical plasmatocytes (arrowheads, compare Fig. 1C,G), corresponding in size to lamellocytes. Crystal cells were not affected by Chn over-expression as melanized crystal cells were still detected in heat-treated *Pxn-GAL4 x UAS-chn* larvae (Fig. S1).

Antibody staining using the lamellocyte marker MAb L1 [31] confirmed that Chn over-expression in plasmatocytes triggered lamellocyte differentiation (Fig. 1D,H), with lamellocyte frequency increasing from 0.5% in the *Pxn-GAL4* driver control to 25.6% (Fig. 1I). Two classes of MAb L1 positive cells were detected. The first corresponded to mature lamellocytes with diameter >40 µm (closed arrowheads in Fig. 1H). The second consisted of cells of diameter between 10–40 µm (open arrowheads in Fig. 1H). These were sized between a typical plasmatocyte and mature lamellocytes and feasibly represent differentiation intermediates between plasmatocytes and mature lamellocytes.

*Pxn-GAL4* is also expressed in crystal cells and at low levels in the fat body [14]. By over-expressing Chn using either the crystal cell-specific driver *lz-GAL4*, or the fat body-specific driver *Lsp2-GAL4* we could exclude these tissues as the source of lamellocytes. Neither *lz-GAL4* nor *Lsp2-GAL4* significantly increased lamellocyte frequency when used to over-express Chn (Fig. 1I, P value >0.05). In contrast, other plasmatocyte-expressed GAL4 drivers were able to induce lamellocyte differentiation when used to over-express Chn. For example, *He-GAL4,* which is expressed in about 30% of all hemocytes [14], increased lamellocyte frequencies to 20.5%, while *Hml-GAL4,* which is expressed in a sub-population of plasmatocytes [32], raised frequencies to 4.9% (Fig. 1I). In *He-GAL4, UAS-GFP x UAS-chn* or *Hml-GAL4, UAS-GFP x UAS-chn* larvae GAL4-mediated expression of GFP is sustained after Chn over-expression. Double staining of hemocytes isolated from these larvae identified cells that were both GFP-positive and MAb L1-positive (Fig. S2), showing that Chn-induced lamellocyte differentiation by these drivers is cell autonomous and that *Hml-GAL4* expressing plasmatocytes can be transformed into lamellocytes. The lower numbers of lamellocytes observed after Chn over-expression using *Hml-GAL4* compared with *Pxn-GAL4* is most likely due to fewer hemocytes expressing *Hml-GAL4* (35%) compared with *Pxn-GAL4* (96%). When lamellocyte number after Chn over-expression is normalized to the number of hemocytes expressing each driver we observed comparable transdetermination rates (Fig. S2).

The ability of Chn over-expression to induce lamellocyte differentiation compared well with other transcription factors and known regulators of lamellocyte differentiation. As shown in Figure 1J, similar lamellocyte frequencies were observed after expression of dominant-negative U-shaped (Ush) variants [22] using *Pxn-GAL4*. However, the transcription factor Kruppel (Kr) and the co-activator Nejire (Nej) which had also been identified in our genetic screen to induce lamellocytes when expressed using *Pxn-GAL4* [14], were much less efficient than Chn, with lamellocyte frequencies of 9.7% and 7.2% respectively observed (Fig. 1J). Significantly, overexpression of the early B cell factor homologue Knot (Kn) failed to induce lamellocyte differentiation, with lamellocyte frequency unchanged from the *Pxn-GAL4* driver alone control (Fig. 1J). Kn was previously shown to be required for lamellocyte development although function is non-cell autonomous [33], a conclusion supported by our results. As an additional control we observed that ectopic expression of another CoREST-interacting transcription factor, Tramtrack88 (Ttk88) [34], failed to induce lamellocyte development (Fig. 1J).

### Pulsed Chn expression increases lamellocyte numbers in circulation

*Pxn-GAL4* is first expressed in plasmatocytes during late embryonic stages [35] and persists during all larval stages [14]. In the previous experiments we examined lamellocyte differentiation at wandering third instar larval stages approximately 100 hrs after the onset of Chn expression in plasmatocytes. To exclude the possibility that lamellocyte induction by Chn was an indirect consequence of long-term expression of Chn we examined the effect of pulsed over-expression of Chn. Precise temporal control of Chn expression was overlaid on *Pxn-GAL4* using the TARGET system [36]. In this approach, *Pxn-GAL4* mediated induction is controlled by ubiquitous expression of a temperature sensitive variant of yeast GAL80 (GAL80ts). GAL80ts represses *Pxn-GAL4* at 18°C, but is inactivated at 29°C, allowing onset of *Pxn-GAL4* mediated expression to be controlled by temperature up-shift (see Fig. S3). This provides a defined time point from which the time course of Chn-induced lamellocyte differentiation can be assayed.

**Figure 2:**
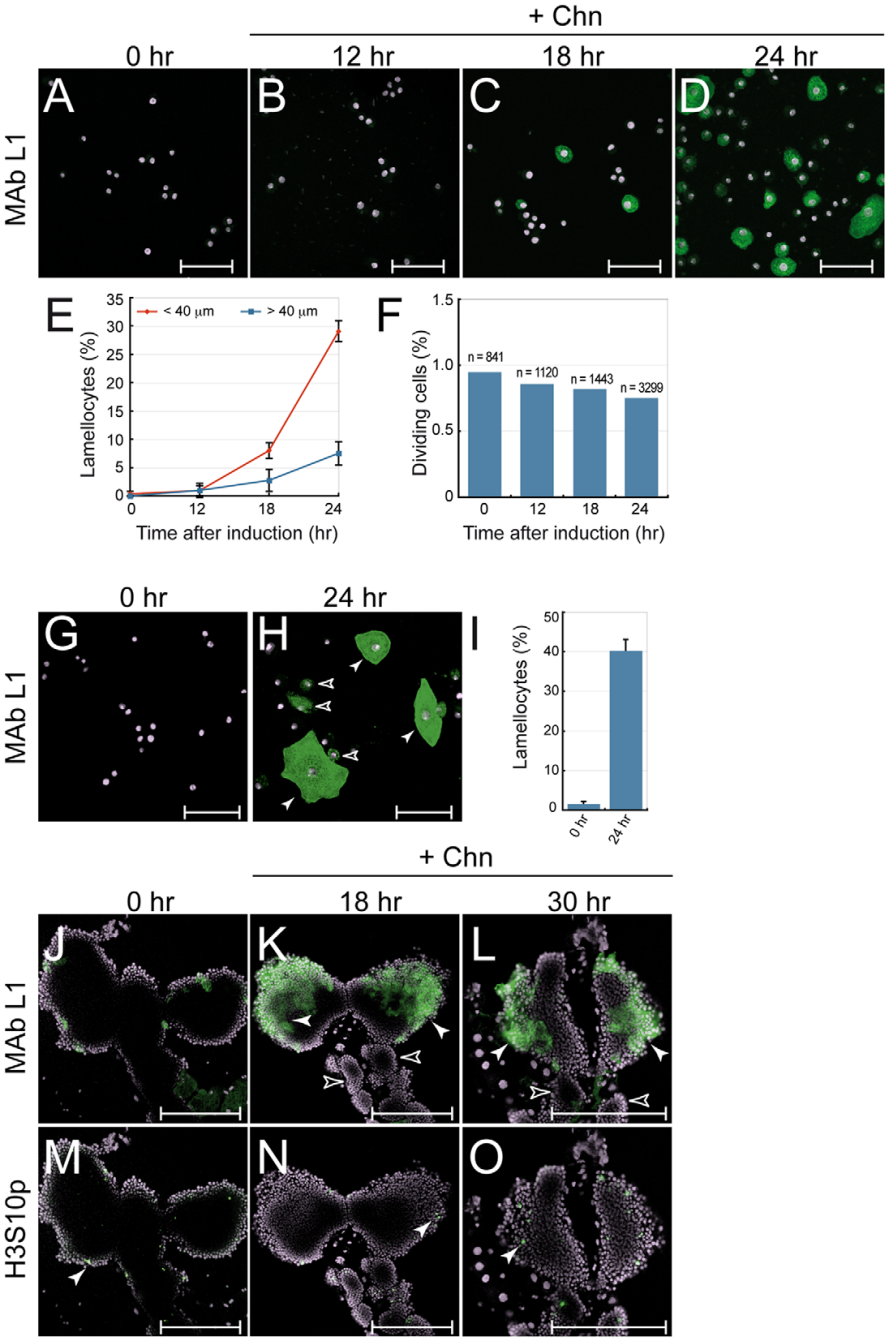
Pulsed Chn overexpression induces lamellocyte differentiation. *Pxn-GAL4*, *UAS-GFP; Ubi-GAL80^ts^ x UAS-chn* wandering stage third instar larvae were raised at 18°C (GAL4 repressed) and Chn was induced by up-shift to 29°C. (**A–D**) Lamellocytes are absent from circulation (**A**) 0 hr and (**B**) 12 hr after induction of Chn expression, but appear at (**C**) 18 hr, peaking at (**D**) 24 hr. (**E**) Proportion of lamellocytes <40 µm (red line) or >40 µm (blue line) relative to total hemocyte number (marked by DAPI staining) Time-points are the mean +/− s.d. of 10 determinations. (**F**) Proliferation index after Chn overexpression determined from number of anti-H3S10p antibody staining hemocytes relative to total hemocyte number (marked by DAPI staining) (**G**) Hemocytes freshly isolated from larvae grown at 18°C do not stain with MAb L1, but (**H**) after in vitro culture at 29°C for 24 hr to induce Chn expression many lamellocytes were detected, including mature (closed arrowheads) and intermediate (<40 µm) (open arrowheads) forms. (**I**) Lamellocyte frequencies before (0 hr) and 24 hr after culture at 29°C. Data are the mean +/− s.d. of 5 independent determinations. (**J–L**) Chn over-expression increases lamellocyte number in primary lymph glands (closed arrowheads), but (**M–O**) does not increase proliferation. Lamellocytes were not detected in the secondary lobes (open arrowheads) In (**J–O**) antibody staining is shown in green, DAPI-stained nuclei in purple. Scalebar in (**A–H**) denotes 50 µm, in (**J–O**) 200 µm.

Larvae were cultured at 18°C until the third larval instar and then shifted to 29°C to induce Chn expression in plasmatocytes. Lamellocytes were rarely seen in larvae 0 hr (Fig. 2A), or 12 hr after up-shift to 29°C (Fig. 2B). However, lamellocyte number increased dramatically at 18 hr (Fig. 2C) and 24 hr (Fig. 2D) after up-shift. The lamellocyte frequency observed at 24 hr was 36.6%, equivalent to that observed after long-term expression of Chn (Fig. 1I). No significant changes in hemocyte proliferation (measured by anti-H3S10p staining) were observed at any point after up-shift to 29°C, with proliferation indexes consistently less than 1% (Fig. 2F), indicating that lamellocyte increase after Chn overexpression was not caused by over proliferation of a small pool of existing lamellocytes or precursors. Moreover the larval lymph glands remained intact over this time course (Figure 2K,L) suggesting that it is unlikely that lamellocytes released into circulation due to fragmentation of the lymph glands account for the observed increases in circulating lamellocyte numbers.

As observed after long term Chn expression, two classes of MAb L1^+^ cells were detected i) mature lamellocytes >40 µm in size and, ii) lamellocytes <40 µm in size, intermediate between plasmatocytes and mature lamellocytes. However, lamellocytes larger than 40 µm showed a lag in appearance relative to those smaller than 40 µm (Fig. 2E). Thus at 24 hr after Chn induction, small MAb L1^+^ cells were 29% of hemocytes, while mature MAb L1^+^ lamellocytes only comprised 6.7%. This is consistent with lamellocytes originating from a small precursor that increases in size as lamellocytes mature.

We next examined whether circulating hemocytes purified from *Pxn-GAL4; GAL80ts x UAS-chn* wandering third instar larvae could differentiate into lamellocytes in isolation in vitro. Circulating hemocytes were isolated from larvae that had been cultured at 18°C. Few of these were MAb L1^+^ lamellocytes (Fig. 2G). However, after culture in vitro at 29°C for 24 hours to initiate Chn over-expression, many MAb L1^+^ lamellocytes were detected (Fig. 2H). Quantitation of lamellocyte frequencies revealed that lamellocyte abundance increased from 1.3% to 40% after Chn induction in vitro (Fig. 2I). These data confirm that lamellocytes detected in circulation after Chn induction can arise from progenitors that exist in circulation prior to Chn over-expression. This is interesting in the light of recent reports that sessile hemocyte compartments have the ability to differentiate into lamellocytes [15]. Our data suggest that this is a common property of hemocytes of embryonic origin, whether in circulation or in sessile compartments.

### Chn triggers lymph gland lamellocyte development

We next tested if this feature is shared by lymph gland plasmatocytes. During larval stages the lymph gland is the site of a second wave of hematopoiesis, and generates hemocytes that are liberated at pupariation [12], [13]. Lymph gland hemocytes have the ability to differentiate into lamellocytes in response to stimuli including, parasitic wasp infestation [9], [12]. To determine whether Chn overexpression can also trigger lymph gland plasmatocytes to form lamellocytes we exploited the normal expression of *Pxn-GAL4* in plasmatocytes and crystal cells in the cortical zone of the lymph gland [13]. As above, temporal control of Chn expression was overlaid over *Pxn-GAL4* using the TARGET (*Ubi-GAL80^ts^*) system. MAb L1 staining revealed that Chn expression in lymph gland plasmatocytes induced the differentiation of large numbers of lamellocytes within 18 hr (Fig. 2K) and 30 hr (Fig. 2L) after up-shift to 29°C. Lamellocytes were detected in the cortical zone of the primary lymph gland lobes (closed arrowheads in Fig. 2K,L). These are the normal sites of plasmatocyte and crystal cell maturation and correspond to the domain of *Pxn-GAL4* expression, indicating that the Chn-induced lamellocyte differentiation is cell autonomous. Importantly, lamellocytes were not observed in secondary lobes that contain undifferentiated prohemocytes (open arrowheads in Fig. 2K,L). Moreover, as noted for circulating hemocytes Chn induction did not increase proliferation of lymph gland hemocytes (Fig. 2M–O). In addition, the larval lymph glands remained intact, thus excluding the possibility of lamellocytes being released into circulation to account for increases in circulating lamellocyte numbers.

### Chn over-expression induces transcription of lamellocyte markers

**Figure 3:**
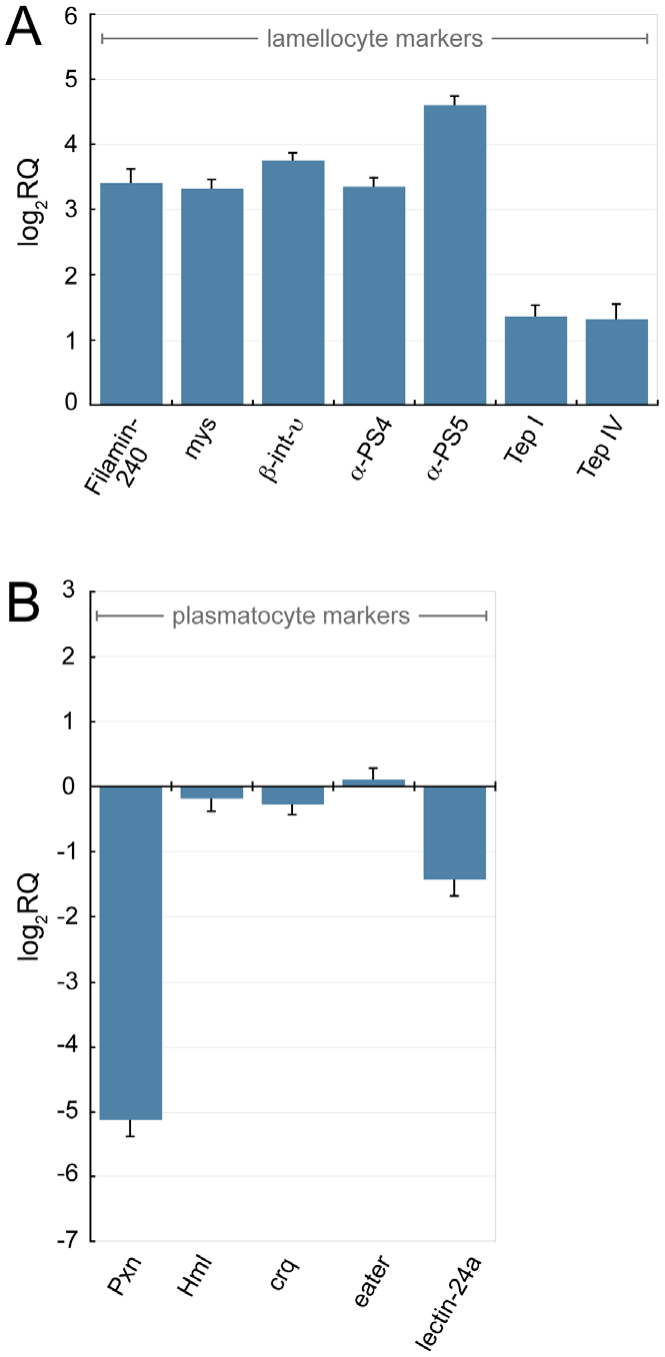
Chn over-expression activates transcription of lamellocyte markers and represses transcription of plasmatocyte markers. *Pxn-GAL4*, *UAS-GFP; Ubi-GAL80^ts^ x UAS-chn* larvae were raised at 18°C (GAL4 repressed) and Chn was induced by up-shift to 29°C for 24 hours. *Pxn-GAL4*, *UAS-GFP; Ubi-GAL80^ts^ x UAS-chn* larvae raised entirely at 18°C provided an uninduced control. mRNA was isolated from hemocytes from wandering stage third instar larvae and relative transcript abundance in Chn expressing relative to uninduced control hemocytes was determined by real-time PCR. *RpL32* provided an endogenous control to normalize expression. Relative transcript abundance after Chn over-expression of (**A**) lamellocyte markers and (**B**) plasmatocyte markers. Data are mean and standard deviation of three independent determinations.

We next confirmed whether Chn over-expression induced expression of other lamellocyte markers. We purified hemocytes from *Pxn-GAL4*, *UAS-GFP*; *Ubi-GAL80^ts^* driver-alone and *Pxn-GAL4*, *UAS-GFP*; *Ubi-GAL80^ts^ x UAS-chn* wandering third instar larvae that had been cultured at 29°C for 24 hours. mRNA was isolated and transcript abundance determined by real-time PCR. We confirmed that transcription of the 240 kDa subunit of Filamin (Filamin-240) is up-regulated 11 fold in hemocytes after Chn induction (Fig. 3A). Filamin-240 has previously been shown to be expressed exclusively on lamellocytes [37]. In a similar manner lamellocytes have previously been shown to stain with antibodies against the β-integrins Myospheroid (Mys) and β^v^-integrin (βInt-v) [19]. Up-regulation of the α-integrins α-PS4 and α-PS5 has also previously been shown to accompany ectopic lamellocyte differentiation observed in *hop^Tum-l^* mutants [19]. We observed that Chn over-expression was accompanied by increased abundance of all these integrins, with *Mys* and *βInt-v* transcript levels increased 10 and 13 fold respectively, and *α-PS4* and *α-PS5* transcript levels increased 10 and 24 fold respectively (Fig. 3A). Thus Chn over-expression is accompanied by increased transcription of lamellocyte markers, confirming data obtained using MAb L1 antibodies.

Conversely, Chn over-expression led to the repression of plasmatocyte markers. Consistent with results observed using the *Pxn-GFP* reporter, Chn over-expression reduced *Pxn* transcription by 35 fold 24 hours after Chn induction (Fig. 3B). Repression of *Pxn* transcription was accompanied by slight reduction in expression of *Croquemort* (*Crq*) and *Hemolectin* (*Hml*), while transcript levels of *eater* were essentially unchanged (Fig. 3B). These transcripts may not be direct targets of Chn and potentially require a longer period of Chn over-expression to observe significant reduction in expression levels.

### Endogenous Chn expression

**Figure 4:**
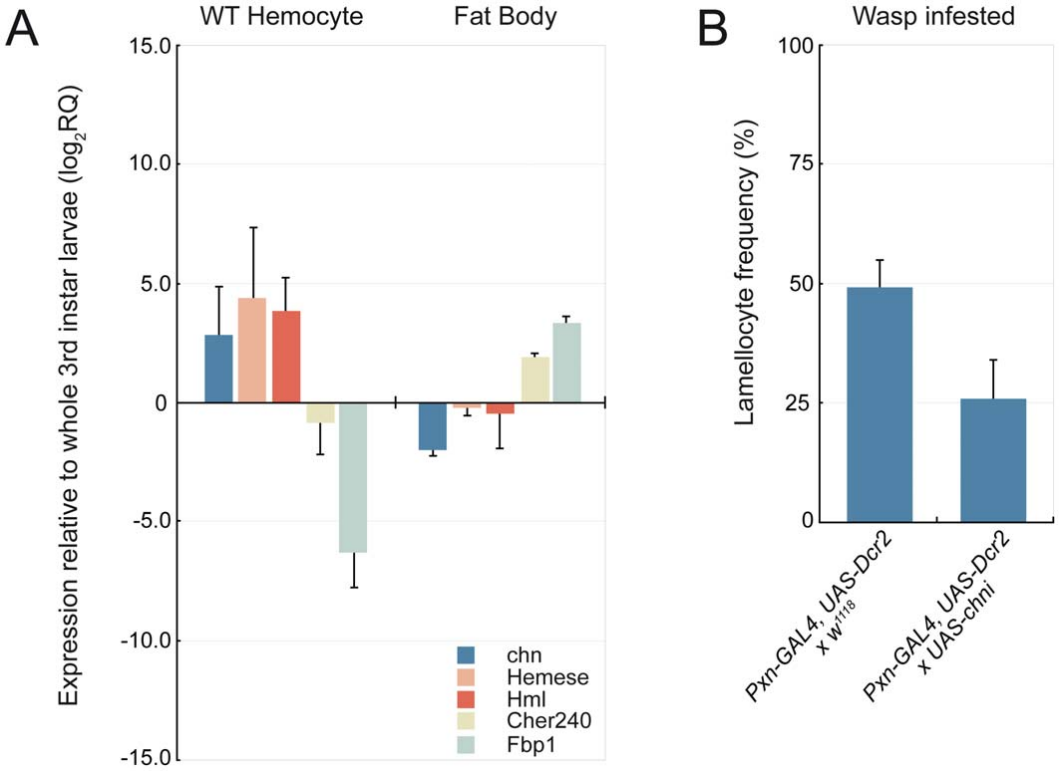
Endogenous Chn expression. (**A**) *chn* transcript levels are elevated in hemocyte populations relative to whole larvae. Elevation of *chn* transcript levels parallels that observed for the hemocyte expressed *He* and *Hml* transcripts. In contrast *chn* transcripts are reduced in fat body relative to whole larvae. Data are mean and standard deviation of three independent determinations (**B**) RNAi knockdown of Chn reduces lamellocyte differentiation observed after wasp infestation. Lamellocyte frequencies were determined after MAb L1 staining. Data are mean and standard deviation of 5 confocal panels from each of 4 separate infested larvae hemocyte preparations.

We next verified whether Chn is normally expressed in larval hemocytes. We compared the relative abundance of *chn* transcripts in purified hemocytes with abundance in whole third instar larvae and isolated fat body tissue. For comparison we assayed expression of hemocyte markers *He* and *Hml* and the fat-body marker *Fat body protein 1* (*Fbp1*). As shown in Figure 4A, *chn* was expressed in wild-type hemocytes and enriched relative to whole third instar larvae 7.5 fold. This was comparable to the observed enrichment of the hemocyte markers *He* and *Hml*. In contrast *chn* expression in fat body was reduced relative to whole third instar larvae. To discriminate whether *chn* may be normally required for lamellocyte differentiation we examined the affect of RNAi knockdown of *chn* transcripts on wasp-induced lamellocyte differentiation. Control *UAS-Dcr-2*; *Pxn-GAL4*, *UAS-GFP x w^1118^* and *UAS-Dcr-2*; *Pxn-GAL4*, *UAS-GFP x UAS-chni* 2^nd^ instar larvae were infested with the parasitic wasp *L. boulardi* G486 for 12 hours and hemocytes isolated 30 hours later and lamellocytes visualized using MAb L1. As shown in Figure 4B, while lamellocyte frequency was almost 50% in control preparations, lamellocyte frequency in *chn* RNAi preparations was reduced to 25%, suggesting that knockdown of *chn* is partially able to block lamellocyte differentiation.

### In vitro differentiation of lamellocytes from plasmatocytes

**Figure 5:**
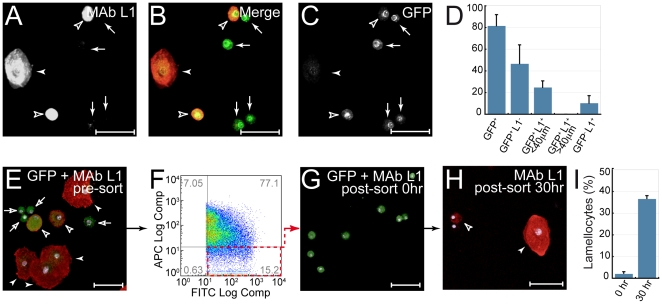
In vitro differentiation of lamellocytes from plasmatocytes. (**A–D**) *hop^Tum-l^/+; Pxn-GAL4-UAS-GFP/+* larvae raised at 20°C contain hemocytes intermediate between mature lamellocytes and plasmatocytes. (**A**) MAb L1 and (**C**) anti-GFP staining. (**B**) Merge showing overlap in MAb L1 (red) and anti-GFP (green) Small (<20 µm) GFP^+^/MAb L1^−^ plasmatocytes (closed arrows), large (>40 µm) GFP^−^/MAb L1^+^ lamellocytes (closed arrowheads) and intermediate sized (<40 µm) GFP^+^/MAb L1^+^ hemocytes (open arrowheads) were detected. (**D**) 24.7% of hemocytes are the intermediate GFP^+^/MAb L1^+^ population. No large (>40 µm) GFP^+^/MAb L1^+^ lamellocytes were detected. Data are mean +/− s.d. of 8 determinations. (**E–I**) FACS purified GFP^+^/MAb L1^−^ plasmatocytes from *hop^Tum-l^/+;* *Pxn-GAL4-UAS-GFP/+* larvae can be induced to differentiate into lamellocytes in vitro at 29°C. (**E**) Hemocytes before sorting, stained with MAb L1 (red) and anti-GFP antibodies (green) Populations are labeled as in (**B**). (**F**) Hemocytes were FACS sorted to select GFP^+^/MAb L1^−^ cells (lower right quadrant) (**G**) Confocal microscopy confirms sorted cells as GFP^+^/MAb L1^−^ plasmatocytes. (**H**) These can differentiate into MAb L1^+^ lamellocytes after in vitro culture at 29°C for 30 hr. Both large mature lamellocytes (closed arrowheads) and (<40 µm) intermediates (open arrowheads) were detected. (**I**) Increase in lamellocyte frequency after culture at 29°C (30 hr) Data are the mean +/− s.d. of three independent determinations. In (**E**,**G**,**H**) DAPI-stained nuclei are shown in purple. Scalebar in all panels denotes 50 µm.

We next examined whether plasmatocytes can be induced to differentiate into lamellocytes in response to other signals. In particular, large numbers of lamellocytes have previously been observed in *hop^Tum-l^* mutants, which encode a constitutively active variant of the *Drosophila* JAK [9], [19]. We used these mutants to explore the transition from Pxn-GFP^+^ plasmatocyte to MAb L1^+^ lamellocyte. Antibody staining of *hop^Tum-l^*; *Pxn-GAL4*, *UAS-GFP* hemolymph discriminated three hemocyte populations. As shown in Fig. 5A–D, GFP^+^/MAb L1^−^ plasmatocytes (arrows), large (>40 µm) GFP^−^/MAb L1^+^ lamellocytes (closed arrowheads), and GFP^+^/MAb L1^+^ hemocytes (open arrowheads) were detected. Large (>40 µm) GFP^+^/MAb L1^+^ lamellocytes were never observed (Fig. 5D). The GFP^+^/MAb L1^+^ hemocytes (open arrowheads) detected were intermediate in size between plasmatocytes (arrows) and mature lamellocytes (closed arrowheads), and resembled the <40 µm MAb L1^+^ lamellocytes observed after pulsed over-expression of Chn. We postulate that these are double-positive intermediates between plasmatocytes and mature lamellocytes.

To provide support for this we purified GFP^+^/MAb L1^−^ plasmatocytes and asked if these can differentiate into lamellocytes in vitro. The *hop^Tum-l^* mutants provide a convenient system to test this, as the mutation is temperature-sensitive conditional. Thus, growth at 20°C weakly activates the JAK/STAT pathway and lamellocyte differentiation, while growth at 29°C induces strong JAK/STAT activation and lamellocyte differentiation. Hemocytes were prepared from *hop^Tum-l^*; *Pxn-GAL4*, *UAS-GFP* larvae grown at 20°C. We confirmed that three hemocyte populations including GFP^+^/MAb L1^−^ plasmatocytes (Fig. 5E, arrows) were present. Cells were sorted on a MoFlo cell sorter to select GFP^+^/MAb L1^−^ plasmatocytes (Fig. 5F, G). This purified plasmatocyte population was then cultured *in vitro* at 29°C to induce fully the JAK/STAT pathway. After 30 hours, both large (>40 µm) MAb L1^+^ lamellocytes (Fig. 5H, closed arrowheads), and smaller (<40 µm) MAb L1^+^ lamellocytes (Fig. 5H, open arrowheads) were detected. The lamellocyte frequency after 30 hours corresponded to 36.6% of cells (Fig. 5I), consistent with the differentiation of purified plasmatocytes into lamellocytes.

### Lineage tracing wasp-induced lamellocytes

**Figure 6:**
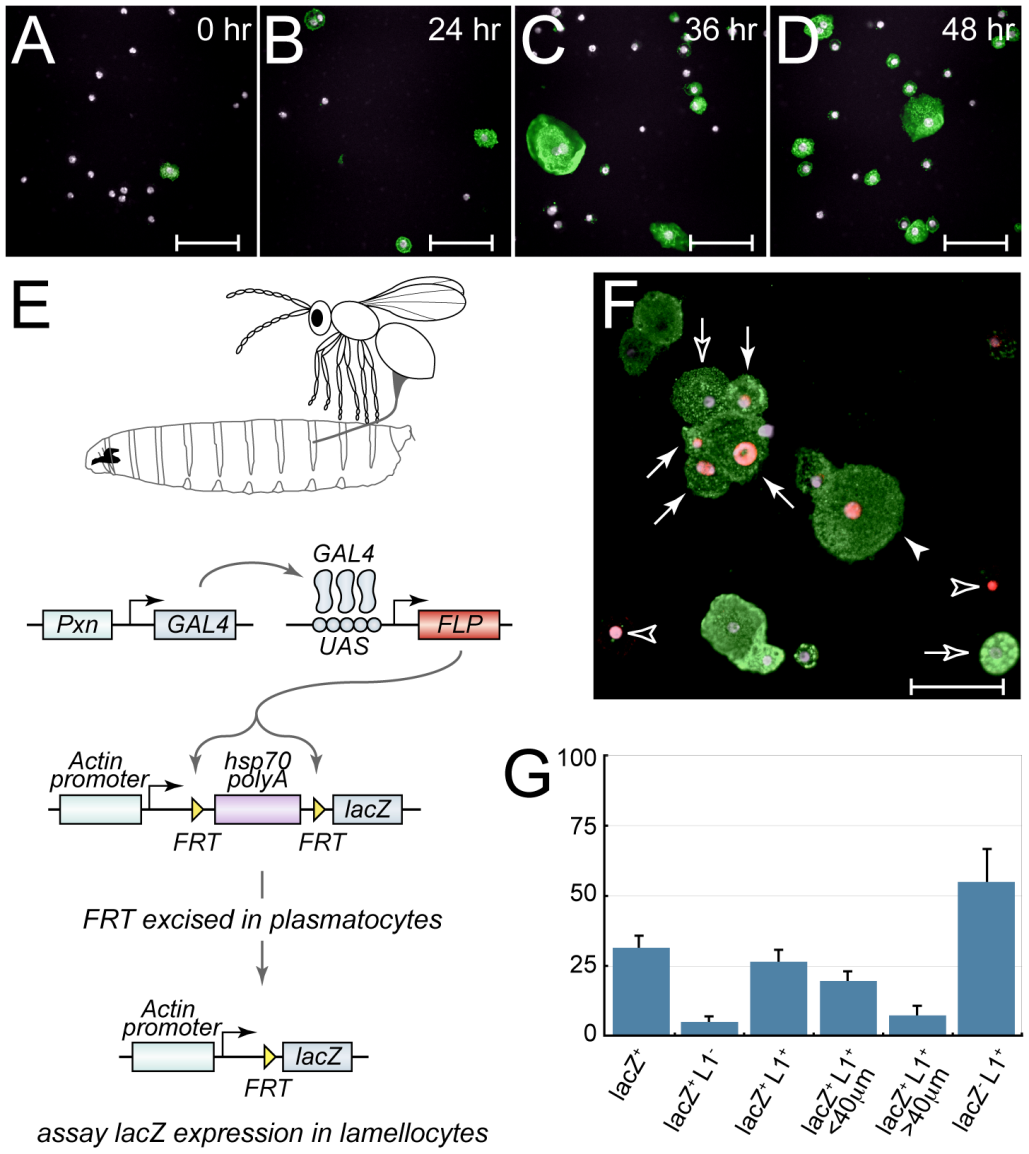
Lineage tracing reveals that wasp-induced lamellocytes arise from plasmatocytes. (**A–D**) MAb L1-staining (green) of hemocytes isolated from third instar larvae (**A**) 0 hr, (**B**) 24 hr, (**C**) 36 hr, and (**D**) 48 hr after 12 hr infestation with the parasitic wasp *L. boulardi.* DAPI-stained nuclei are shown in purple. Scalebar denotes 50 µm. (**E**) Schematic of the lineage tracing experiment. *Pxn-GAL4* drives expression of FLP in plasmatocytes. FLP excises an FRT flanked transcription stop cassette from the *act5C>stop>lacZ* transgene, turning on expression of lacZ in a plasmatocytes. *act5C-lacZ* expression is then independent of *Pxn-GAL4*, marking cells that were plasmatocytes during subsequent lamellocyte differentiation. (**F**) MAb L1 (green) and anti-lacZ (red) antibody staining reveals lacZ^+^/MAb L1^+^ lamellocytes confirming the plasmatocyte origin of lamellocytes. Three classes of lamellocytes occur, lacZ^−^ lamellocytes (open arrows), and lacZ^+^ lamellocytes that are <40 µm (closed arrows) or >40 µm (closed arrowheads) lacZ^+^/MAb L1^−^ hemocytes indicated by open arrowheads. (**G**) 31.3% of hemocytes are lacZ^+^, of which 26.4% are lacZ^+^/MAb L1^+^. Consistent with the origin of lamellocytes from small precursors, lacZ^+^/MAb L1^+^ cells <40 µm in diameter and cells >40 µm in diameter are detected.

Lamellocyte differentiation induced by de-regulated activation of the JAK/STAT pathway or over-expression of Chn could be argued to be synthetic phenotypes. To confirm if lamellocytes differentiate from plasmatocytes in response to natural stimuli, we infested larvae with the parasitic wasp *L. boulardi* G486. *Drosophila* larvae were infested with female wasps for 12 hours, and circulating lamellocyte differentiation was followed 0, 24, 36 and 48 hours after infestation. We observed robust differentiation of circulating lamellocytes 36 and 48 hours after infestation (Fig. 6C, D). To determine whether wasp-induced lamellocytes derive from plasmatocytes we performed a lineage tracing experiment using the *act5C>stop>lacZ* flip-out system (Fig. 6E, [38]). We isolated circulating hemocytes from third instar larvae 36 hours after infestation and stained using MAb L1 and anti-lacZ antibodies. *Pxn-GAL4*-mediated FLP expression activated lacZ-expression in 31.3% of hemocytes (Fig. 6G). Although the frequency of lacZ^+^ cells was lower than expected, as *Pxn-GAL4* is expressed in 95% of hemocytes [14], it was sufficient to trap lacZ^+^/MAb L1^+^ lamellocytes (Fig. 6F), including mature lamellocytes (closed arrowheads) and intermediates (closed arrows). Quantitation revealed that 26.4% of hemocytes were lacZ^+^/MAb L1^+^, of which 19.4% were intermediates (<40 µm), and 7.2% were the typical large lamellocyte form (Fig. 6G). lacZ^−^/MAb L1^+^ lamellocytes (open arrows) and lacZ^+^/MAb L1^−^ hemocytes (open arrowheads) were also detected.

### Phagocytes can differentiate into lamellocytes

**Figure 7:**
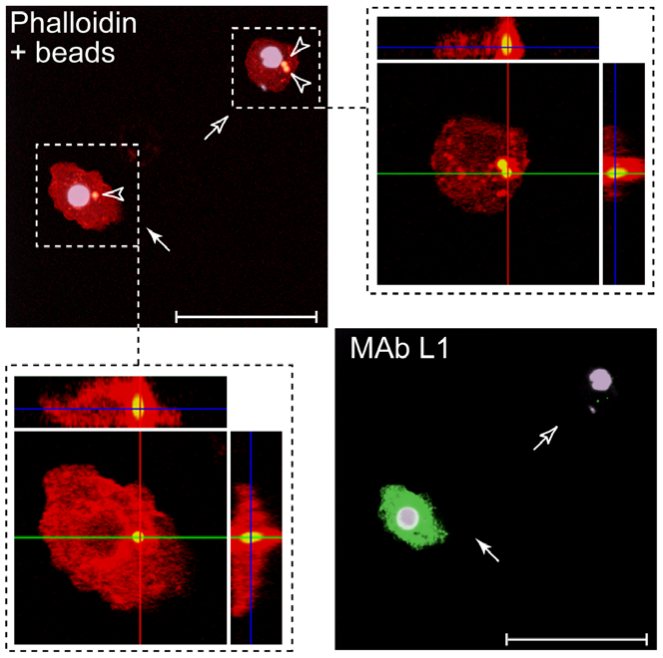
Active phagocytes can differentiate into lamellocytes. *Pxn-GAL4*, *UAS-GFP; Ubi-GAL80^ts^ x UAS-chn* larvae were raised at 18°C (GAL4 repressed) and injected with fluorescent polystyrene microspheres. Chn expression was induced by up-shift to 29°C for 24 hours to trigger lamellocyte differentiation. Isolated hemocytes were stained using MAb L1 antibodies to identify lamellocytes, and using rhodamine-phalloidin to distinguish cell boundaries. Engulfed microspheres were identified by intense yellow green fluorescence (which bleeds through into the red channel). A confocal Z stack was compiled through a MAb L1^−^ plasmatocyte and a MAb L1^+^ lamellocyte. Projections along the X- and Y-axis confirmed that both contained engulfed microspheres. In panels rhodamine-phalloidin staining is shown in red, fluorescent polystyrene microspheres as yellow spots (open arrowheads) and MAb L1 staining is pseudocolored green. MAb L1^+^ lamellocyte is indicated by a closed arrow and MAb L1^−^ plasmatocyte is indicated by an open arrow.

Finally we examined whether plasmatocytes that differentiate into lamellocytes are naïve, or whether plasmatocytes that have been conditioned by challenge may also differentiate into lamellocytes. To do this, we tested whether plasmatocytes that are active phagocytes can differentiate into lamellocytes. *Pxn-GAL4*, *UAS-GFP*; *Ubi-GAL80^ts^ x UAS-chn* larvae were cultured at 18°C. Larvae were injected with fluorescent polystyrene microspheres and allowed to recover at 18°C for 2 hours. During the recovery period, the microspheres are actively engulfed by phagocytically active plasmatocytes, marking these plasmatocytes during subsequent analysis. After the recovery period, larvae were shifted to 29°C for 24 hrs to induce lamellocyte differentiation. Hemocytes were then isolated and stained using MAb L1 antibodies to detect lamellocytes and rhodamine phalloidin to reveal cell boundaries. As shown in Figure 7, confocal microscopy detected internalized beads in MAb L1^−^ plasmatocytes (open arrows). Internalized beads could also be detected in MAb L1^+^ lamellocytes (closed arrows), suggesting that these originated from phagocytically active cells.

## Discussion

*Drosophila* provide a genetically tractable model system to investigate cellular innate immune function [1]–[3]. In this report we have examined the origins of lamellocytes, which are *Drosophila* hemocytes that differentiate in response to parasite infestation. We show here that over-expression of the CoREST-interacting transcription factor Chn in plasmatocytes induces lamellocyte differentiation, both in circulation and in lymph glands. Our data indicate that Chn over-expression transforms plasmatocytes into lamellocytes. Consistent with this, we are able to detect double-positive intermediates between plasmatocytes and lamellocytes, and show that isolated plasmatocytes in vitro can be triggered to differentiate into lamellocytes following Chn over-expression. This property is not limited to Chn as we observed that other stimuli, including activation of the JAK/STAT pathway and the natural response to parasitic wasp infestation, also induced lamellocyte formation from plasmatocytes.

Our data suggest that Chn may control lamellocyte development. Previously defined regulators of lamellocyte development include the transcription factor STAT92E, the FOG-1 homologue Ush, and the NURF chromatin remodelling complex. STAT92E functions as an inducer of lamellocyte development, as gain-of-function *hop^Tum-l^* mutants that activate the JAK/STAT pathway cause lamellocyte over-production. In contrast, both loss-of-function *ush* and *Nurf* mutants exhibit increased lamellocyte numbers [19], [22]. Like the homologous FOG-1-GATA-1 pairing, Ush modulates activity of the *Drosophila* GATA factor Srp [39] to favour plasmatocyte differentiation [22]. Recent data in mammalian systems indicates that FOG-1 mediates its effect on GATA-1 in part via recruitment of the transcriptional co-repressor NURD [40], suggesting that Ush functions similarly to repress expression of gene targets required for lamellocyte differentiation in plasmatocytes. Likewise, NURF also inhibits lamellocyte differentiation, in this case by preventing activation of targets of the JAK/STAT pathway [19].

The current biochemical data suggest that Chn is a transcription repressor as Chn recruits the co-repressor complex CoREST [26]. Indeed Tsuda and colleagues have shown that Chn over-expression represses Delta expression in the eye imaginal disk, while we have shown that Chn over-expression is accompanied by repression of some plasmatocyte markers. However, we have also shown that Chn over-expression leads to elevated expression of lamellocyte markers, and Escudero and colleagues [25] have demonstrated that Chn over-expression increases expression of the proneural genes Achaete and Scute. Our data do not allow us to discriminate whether Chn functions entirely as a transcriptional repressor or whether it may also activate transcription. However, the temporally-controlled Chn induction system (*Pxn-Gal4* TARGET) that we have utilized here will allow the primary gene targets of Chn to be determined. By analyzing transcriptional profiles of hemocytes at defined time points after Chn over-expression the primary responders to Chn over-expression will be able to be identified. It will be possible to discriminate whether these targets are preferentially activated or repressed, and also subsequently determine recruitment of transcription co-activator or co-repressor complexes such as CoREST at these targets using chromatin immunoprecipitation (ChIP).

**Figure 8:**
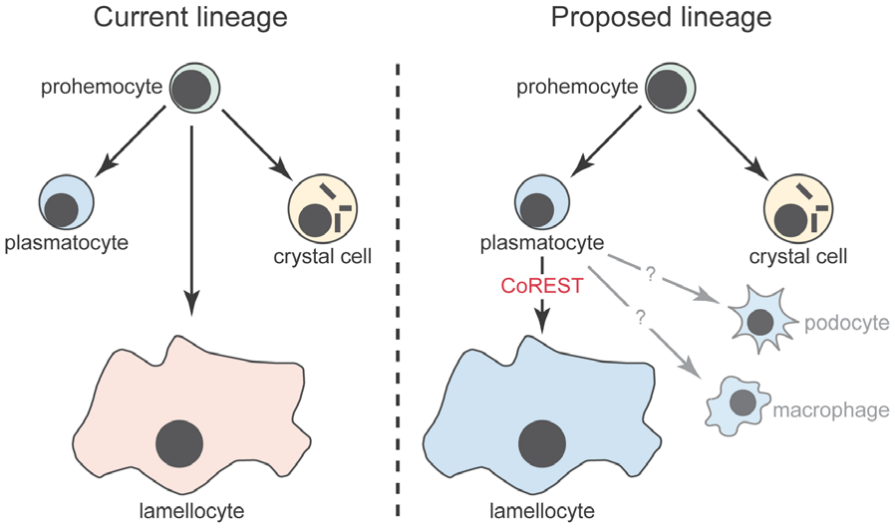
Proposed model of hemocyte lineage. Current models for the origin of the three hemocyte types propose that plasmatocytes, crystal cells and lamellocytes represent distinct lineages that arise separately from a common stem cell or prohemocyte. We propose that prohemocytes generate either crystal cells or plasmatocytes. Plasmatocytes are a plastic population that can, in turn, generate other hemocyte types such as lamellocytes and podocytes.

Our data demonstrating that lamellocytes can originate from plasmatocytes sheds new light on hemocyte lineages. As shown in Figure 8, current models of hemocyte lineages speculate that plasmatocytes, crystal cells and lamellocytes are distinct lineages that arise separately from a common stem cell or prohemocyte [1]–[3], [12]. We propose, however, that prohemocytes generate either crystal cells or plasmatocytes. We suggest that plasmatocytes are a plastic population that can generate other frequently observed hemocyte types including lamellocytes. This model is strikingly reminiscent of the initial hemocyte lineages first proposed by Rizki in 1957 [41]. According to Rizki, prohemocytes were predicted to generate either crystal cells or plasmatocytes, with plasmatocytes differentiating further into activated cells (podocytes) and then lamellocytes [41]. This model has support from a number of experimental studies including this manuscript. Foremost amongst these are recent studies of hemocyte functions of Ush. Dominant-negative Ush variants are able to induce lamellocyte differentiation and it has been suggested that Ush regulates lamellocyte differentiation from a potential plasmatocyte [22]. Secondly, lamellocyte differentiation in response to *Salmonella* infection is blocked in *decapentaplegic* mutants with a corresponding increase in plasmatocyte number, suggesting that lamellocytes arise from plasmatocytes or a common precursor [23].

Two recent studies also suggest that plasmatocytes are a plastic population that may be able to differentiate into lamellocytes. Marking of embryonic plasmatocytes using the *gcm-GAL4* or *sn-GAL4* drivers and an *act5C>stop>GAL4* flip-out transgene shows that lamellocytes that arise in larvae after wasp infestation may originate from cells that had expressed *gcm-GAL4* or *sn-GAL4* in embryos [42]. Similar results have also been observed using the *act5C>stop>GAL4* flip-out transgene and *Pxn-GAL4* and *eater-GAL4* [43]. In both these cases the elicitor of the FLP/FRT activation event and the subsequent sustained marker are the same, namely GAL4 expression. However, in our lineage tracing experiments, GAL4 expression initiates the FLP/FRT activation of a distinct marker, lacZ protein. These data, taken together with our lineage tracing experiments and in vitro differentiation studies suggest that the plasmatocyte is an inherently plastic cell type that is capable of being reprogrammed to tailor immune responses to suit the infectious threats faced by the host. In humans, lymphocyte and leukocyte plasticity [44]–[46] has a significant impact on immune responses. An important future challenge is to establish the full spectrum of *Drosophila* plasmatocyte heterogeneity and exploit the utility of the *Drosophila* genetic system to dissect the mechanisms that regulate such leukocyte plasticity.

## Materials and Methods

### Fly strains and genetic crosses

The following GAL4 drivers were used: *w^1118^*; *Pxn-GAL4*, *UAS-GFP* [35]; *Hml-GAL4* [32]; *lz-GAL4* [42]; *Lsp2-GAL4*; *He-GAL4* [30]. UAS lines used were: *UAS-chn* [25]; *UAS-ush-F13* (aa 302-1191) [22]; *UAS-kn* [17]; *UAS-nej* [45]; *UAS-ttk88* [34] and *UAS-Kr* [46]. For crosses, 5–10 driver line virgin females were mated with 5 males of each UAS strain. Progeny larvae were staged and analyzed as described in [14] and processed for confocal immunofluorescence microscopy as described below.

Temporal control of GAL4-mediated Chn expression was using the TARGET system [36]. The *w^1118^*; *Pxn-GAL4*, *UAS-GFP*; *GAL80^ts^* driver was generated by crossing *w^1118^*; *Pxn-GAL4*, *UAS-GFP*; *+/+* with *w^1118^*; *+/+*; *GAL80^ts^ (w*; *P{tubP-GAL80^ts^}2).* Virgin driver females were crossed with *UAS-chn* males and crosses maintained at 18°C to repress GAL4. Progeny were shifted to 29°C to inactivate GAL80 and induce *Pxn-GAL4*-mediated Chn expression.

### Immunofluorescence microscopy

Circulating hemocytes were isolated from five staged wandering third instar larvae and processed for immunofluorescence microscopy using multispot slides (PH-001, C.A.Hendley) as described in [14]. Confocal microscopy was using a Zeiss Axiovert 100 M. Antibodies used were: chicken anti-GFP antibody (1∶400, Upstate Biotechnology), mouse MAb L1b (1∶60, [31]), mouse MAb H2 anti-Hemese (1∶50, [30]), rabbit anti-β-galactosidase (1∶2000, Cappel), mouse MAb 40-1a anti-β-galactosidase (1∶100), rabbit anti-Histone H3 phospho S10 (H3S10p; 1∶2000, Abcam ab5176). Secondary antibodies from Jackson Immunoresearch were: Cy3-conjugated anti-mouse IgG (H+L), FITC-conjugated anti-chicken IgY (H+L), and Cy3-conjugated anti-rabbit IgG (H+L), all used at 1∶1000.

### Chn induction in hemocytes *in vitro*

Virgin *w^1118^*; *Pxn-GAL4*, *UAS-GFP*; *GAL80^ts^* females were crossed with *UAS-chn* males. Crosses and progeny were maintained at the non-restrictive temperature of 18°C to maintain repression of GAL4. Hemocytes were isolated from 50 wandering stage third instar *Pxn-GAL4, UAS-GFP; GAL80^ts^ x UAS-chn* larvae that had been cultured at 18°C. Hemocytes were centrifuged at 260 *g* for 3 minutes at 4°C and resuspended in 500 µl of Schneider's Drosophila medium (Gibco) containing 10% fetal bovine serum (Invitrogen). 1/5 of the cell suspension was fixed and stained immediately with MAb L1 antibodies. The remaining cell suspension was cultured in 8 well chamber slides (Labtek II) at 29°C for 24 hr. Hemocytes were fixed in 4% paraformaldehyde at room temperature for 10 minutes and stained with MAb L1 antibodies.

### Marking phagocytically active plasmatocytes

Phagocytosis was monitored by injection of fluorescent polystyrene microspheres (Yellow-green 2.0 µm Fluospheres, Invitrogen (F8827)) into the hemocoel of third instar larvae. Fluospheres were vortexed vigorously to ensure even suspension and washed in PBS containing 0.1% BSA (Upstate). Beads were transferred to a drawn glass injection needle (World Precision Instruments, TW100F-4). *w^1118^*; *Pxn-GAL4*, *UAS-GFP*; *GAL80^ts^ x UAS-chn* early third instar larvae were immobilized on double-sided tape (Scotch 665, 3 M) attached to a glass slide. Larvae were injected with fluospheres using an Eppendorf Femtojet microinjector and Leitz Labovert inverted microscope with injection stand and needle holder (Leitz). Injections were made at the posterior of the larvae. Larvae were incubated for 2 hours at room temperature to recover and then transferred to 29°C for 24 hours to induce Chn expression. Hemocytes were prepared for microscopy and stained as described above.

### Visualizing crystal cells

Third instar larval crystal cells were visualized by incubating larvae in 1X PBS for 5 minutes at 60°C. Black crystal cells were observed using an Olympus SZX12 stereo microscope.

### Cell sorting

*hop^Tum-l^/+*; *Pxn-GAL4*, *UAS-GFP/+* larvae were maintained at 20°C. Hemocytes were isolated from 750 larvae in batches of 50 as described [19]. Hemocytes were centrifuged at 260 *g* for 3 minutes at 4°C and washed twice in ice cold PBS. Hemocytes were stained with MAb L1 (1∶60) for 30 minutes on ice, washed three times in ice cold PBS and stained with biotinylated anti-mouse IgG (H+L) antibodies (Jackson Immunoresearch, 1∶1000) for 30 minutes on ice. After three washes with ice cold PBS, hemocytes were stained with APC-streptavidin (BD Pharmingen, 1∶750) for 30 minutes on ice, and washes repeated. Cells were filtered through a CellTrics 50 µm filter (Partec) before sorting on a MoFlo high-speed cell sorter (Beckman Coulter). Hemocytes from 100 *hop^Tum-l^/+*; *Pxn-GAL4*, *UAS-GFP/+* larvae processed as above but not stained with MAb L1 served as a GFP only control. Hemocytes from 100 *w^1118^* larvae processed as above but only stained with biotinylated anti-mouse IgG (H+L) and APC-streptavidin provided a secondary antibody only control to determine baseline APC. Cells were first gated on forward scatter versus side scatter to select viable cells, then on pulse width versus forward scatter to remove doublets, thirdly on compensated FITC signal to select GFP-positive hemocytes, and finally on compensated APC to select MAb L1-negative cells. Gates were set to select GFP-positive MAb L1-negative hemocytes. Hemocytes were sorted into Schneider's Drosophila medium (Gibco, 11720) containing 10% fetal bovine serum (Invitrogen) and cultured in 8 well chamber slides (Labtek II) at 29°C for 30 hours. Hemocytes were subsequently fixed with 4% paraformaldehyde at room temperature for 10 minutes and stained with MAb L1 antibodies and processed for confocal microscopy as described above.

### Wasp infestation

Lineage tracing experiments were performed by crossing virgin females of the genotype *y^1^, w^1118^*, *UAS-Flp.Exel*; *act5C>stop>lacZ* with *w^1118^*; *Pxn-GAL4*, *UAS-GFP* males and the progeny maintained at 25°C. Late second instar/early third instar larvae were infested with 5 females of *Leptopilina boulardi* G486 at 25°C for 12 hours. Hemocytes were prepared from larvae aged for 30 hours and stained using MAb L1 and anti-lacZ antibodies.

RNAi-mediated knockdown of Chn was achieved by crossing *w^1118^, P{UAS-Dcr-2.D}1; Pxn-GAL4, UAS-GFP* virgin females with *UAS-chni* line 35507 provided by the Vienna Drosophila RNAi Center. Control (driver crossed with *w^1118^* males) and knockdown 2^nd^ instar larvae were infested with *L. boulardi* as described above, aged for 30 hours and lamellocytes visualized using MAb L1.

### Real-time PCR analysis of transcript levels

Hemocytes were isolated from a total of 300 third instar larvae of each genotype in batches of fifty animals. Larvae were bled into HyQ CCM3 culture media containing protease inhibitors and hemocytes pelleted by centrifugation at 260 *g* for 5 minutes at 4°C. Hemocytes were washed twice with PBS. mRNA was isolated from hemocytes using a µMACS mRNA Isolation kit (Miltenyi Biotec Ltd, UK) according to the manufacturers instructions. For analysis of transcript abundance in third instar larvae, mRNA was isolated from 5 *w^1118^* larvae using µMACS mRNA Isolation kit. cDNA was generated by reverse transcription using Superscript II (Invitrogen) at 42°C. Real-time PCR was performed on an Applied Biosystems Step One Plus real-time PCR machine. Reactions were performed using Absolute QPCR SYBR green ROX mix (Thermo Fisher Scientific, AB-1162). Primers used are described in Table S1. *RpL32* transcript provided the endogenous control for normalization.

## Supporting Information

Figure S1 Chn over-expression doe not affect crystal cell development. (A) *Pxn-GAL4*, *UAS-GFP x w1118* and (B) *Pxn-GAL4, UAS-GFP x UAS-chn* third instar larvae were heat-treated to reveal crystal cells (black cells in right panels). Chn over-expression disrupts crystal cell accumulation at sessile compartments but does not decrease crystal cell number.
(4.36 MB TIF)

Figure S2 Chn induced-lamellocyte differentiation is cell autonomous. Hemocytes isolated from (A) *Hml-GAL4*, *UAS-GFP/UAS-chn* and (B) *He-GAL4*, *UAS-GFP/UAS-chn* third instar larvae were stained with MAb L1 (red) and anti-GFP (green) antibodies. Nuclei were also stained using DAPI (shown in purple). Hemocytes that are both GFP- and MAb L1-positive could be detected (closed arrowheads). GFP-positive hemocytes that do not stain with MAb L1 are indicated (open arrowheads). Scalebar represents 50 µm. (C) Lamellocyte frequencies observed after Chn expression using *Pxn-GAL4* and *Hml-GAL4* were normalized to the percentage of hemocytes that express GAL4 in the *Pxn-GAL4* and *Hml-GAL4* driver lines. The resultant ratio (transdetermination frequency) shows that *Pxn-GAL4* and *Hml-GAL4* have similar abilities to induce lamellocyte differentiation.
(0.92 MB TIF)

Figure S3 Control of *Pxn-GAL4* mediated expression by GAL80^ts^. (A,B) Schematic illustrating the TARGET system. (A) A ubiquitously expressed, temperature-sensitive GAL80 variant (GAL80^ts^) is functional at 18°C. GAL80^ts^ binds to and represses GAL4 expressed by *Pxn-GAL4* in plasmatocytes, to repress GAL4-mediated transcription of responders like *UAS-GFP*. (B) At 29°C, GAL80^ts^ is inactivated and GAL4 expressed by *Pxn-GAL*4 in plasmatocytes can activate transcription of targets like *UAS-GFP*. (C-H) (GAL80^ts^) can regulate *Pxn-GAL4* mediated gene expression in hemocytes. Hemocytes were isolated from (C) *Pxn-GAL4*, *UAS-GFP* third instar larvae, (D) *Pxn-GAL4*, *UAS-GFP*; *GAL80^ts^* third instar larvae raised 18°C, and (E) *Pxn-GAL4*, *UAS-GFP*; *GAL80^ts^* third instar larvae raised at 18°C but shifted to 29°C for 24 hr. Hemocytes were stained with anti-GFP antibodies (green) and DAPI (blue) to reveal nuclei. GFP is detected in *Pxn-GAL4*, *UAS-GFP* hemocytes, and *Pxn-GAL4*, *UAS-GFP*; *GAL80^ts^* hemocytes at 29°C, but is not detected in hemocytes from *Pxn-GAL4*, *UAS-GFP*; *GAL80^ts^* larvae raised at 18°C. GFP expression in (C) and (E) is equivalent. (F–H) Live intact larvae of the equivalent genotypes to (C–E) showing GFP fluorescence in dorsal sessile hemocyte compartments (dashed ellipse). (F) GFP-expressing hemocytes are detected in sessile compartments in *Pxn-GAL4*, *UAS-GFP larvae*. (G) The sessile patches are indistinguishable from the surrounding tissues at 18°C but (H) become clearly visible after 24 hr temperature upshift at 29°C.
(4.40 MB TIF)

Table S1 Primers used for real-time PCR analysis.
(0.10 MB DOC)
