# Supplementary figures and images for: Lineage Tracing of Lamellocytes Demonstrates Drosophila Macrophage Plasticity
Source: PLoS One. 2010 Nov 19;5(11):e14051. doi: 10.1371/journal.pone.0014051 (PMC2988793; doi:10.1371/journal.pone.0014051)

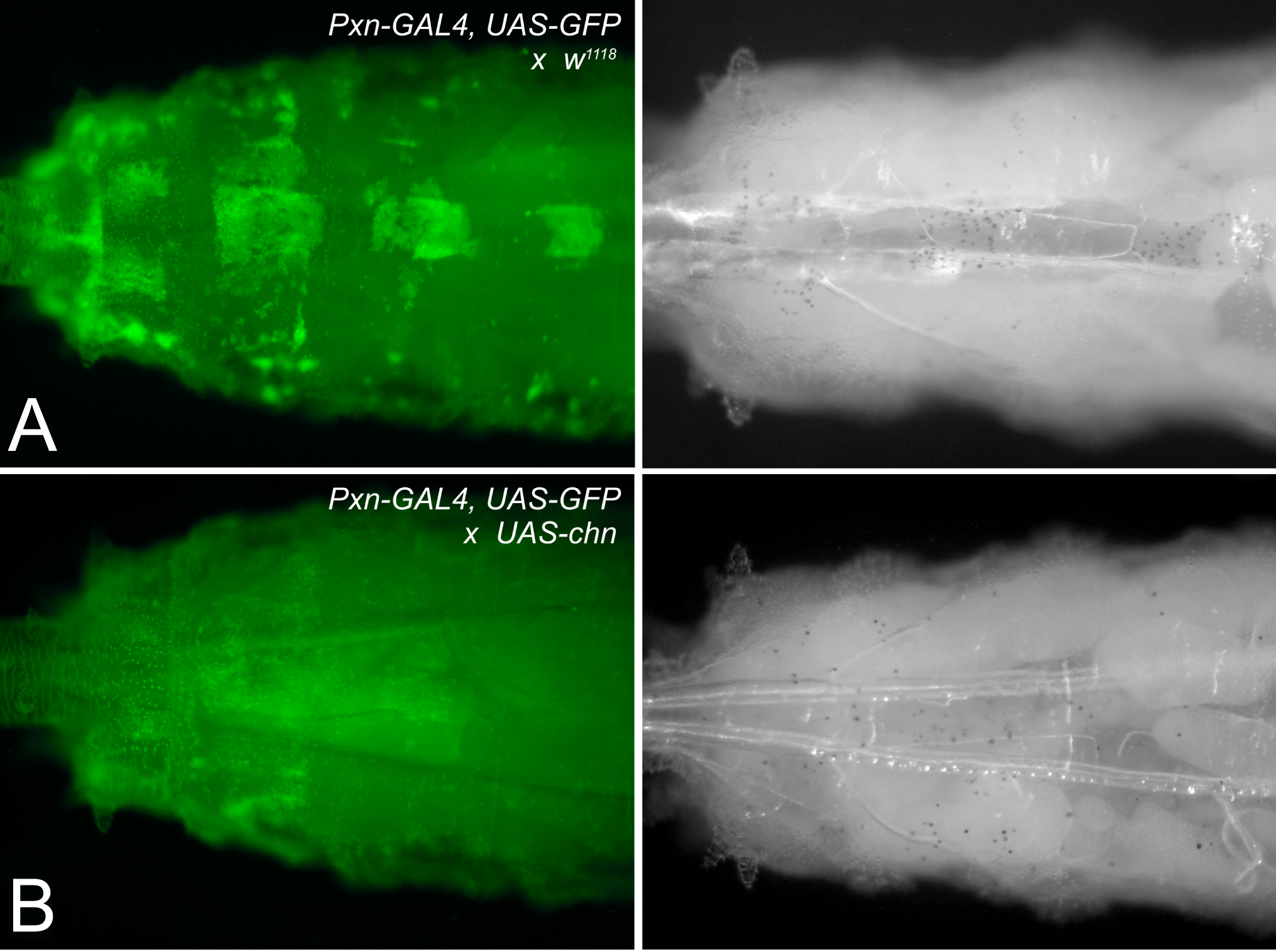

Supplement: Figure S1 — Chn over-expression doe not affect crystal cell development. (A) Pxn-GAL4, UAS-GFP x w1118 and (B) Pxn-GAL4, UAS-GFP x UAS-chn third instar larvae were heat-treated to reveal crystal cells (black cells in right panels). Chn over-expression disrupts crystal cell accumulation at sessile compartments but does not decrease crystal cell number. (4.36 MB TIF) [file pone.0014051.s001.tif]

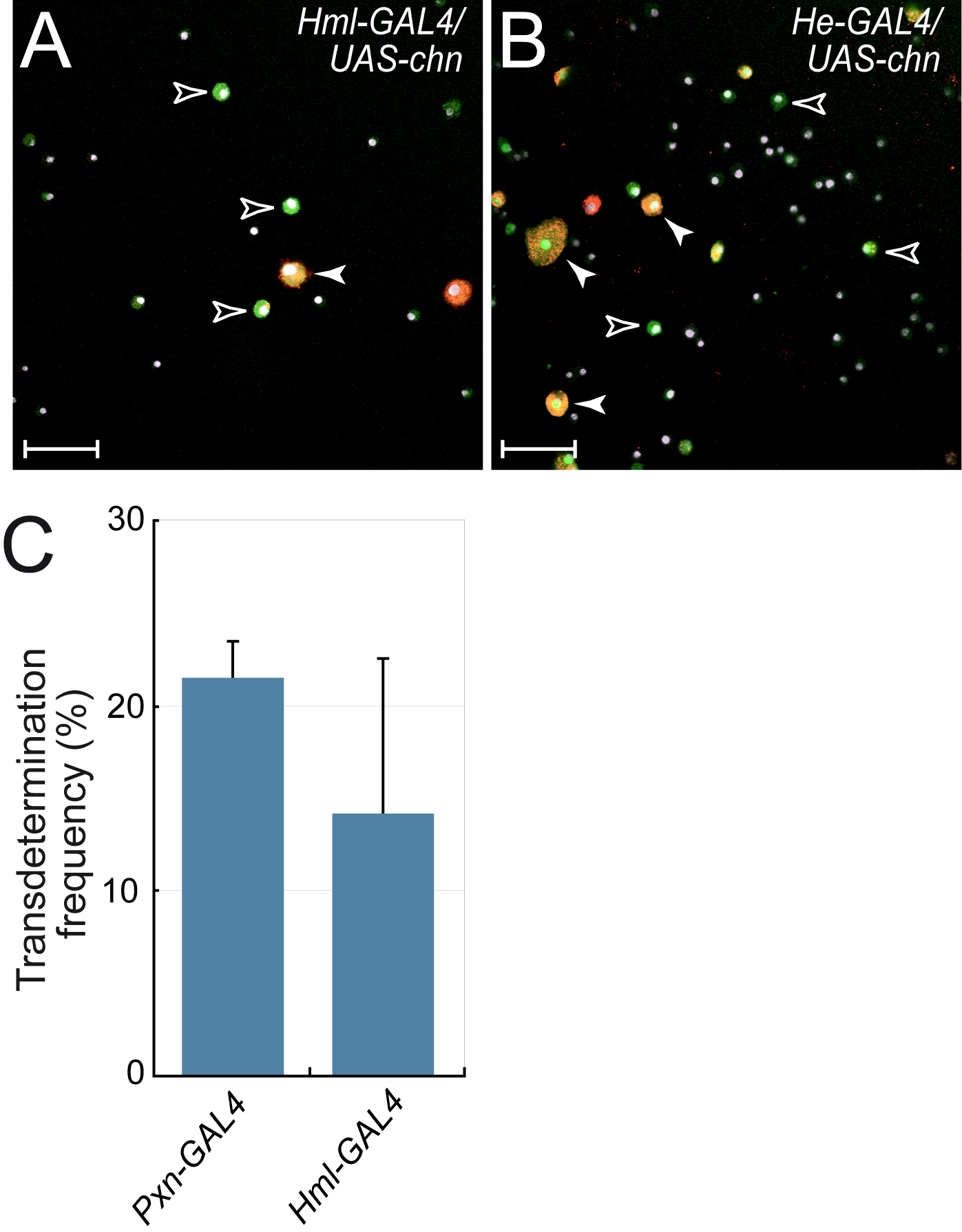

Supplement: Figure S2 — Chn induced-lamellocyte differentiation is cell autonomous. Hemocytes isolated from (A) Hml-GAL4, UAS-GFP/UAS-chn and (B) He-GAL4, UAS-GFP/UAS-chn third instar larvae were stained with MAb L1 (red) and anti-GFP (green) antibodies. Nuclei were also stained using DAPI (shown in purple). Hemocytes that are both GFP- and MAb L1-positive could be detected (closed arrowheads). GFP-positive hemocytes that do not stain with MAb L1 are indicated (open arrowheads). Scalebar represents 50 µm. (C) Lamellocyte frequencies observed after Chn expression using Pxn-GAL4 and Hml-GAL4 were normalized to the percentage of hemocytes that express GAL4 in the Pxn-GAL4 and Hml-GAL4 driver lines. The resultant ratio (transdetermination frequency) shows that Pxn-GAL4 and Hml-GAL4 have similar abilities to induce lamellocyte differentiation. (0.92 MB TIF) [file pone.0014051.s002.tif]

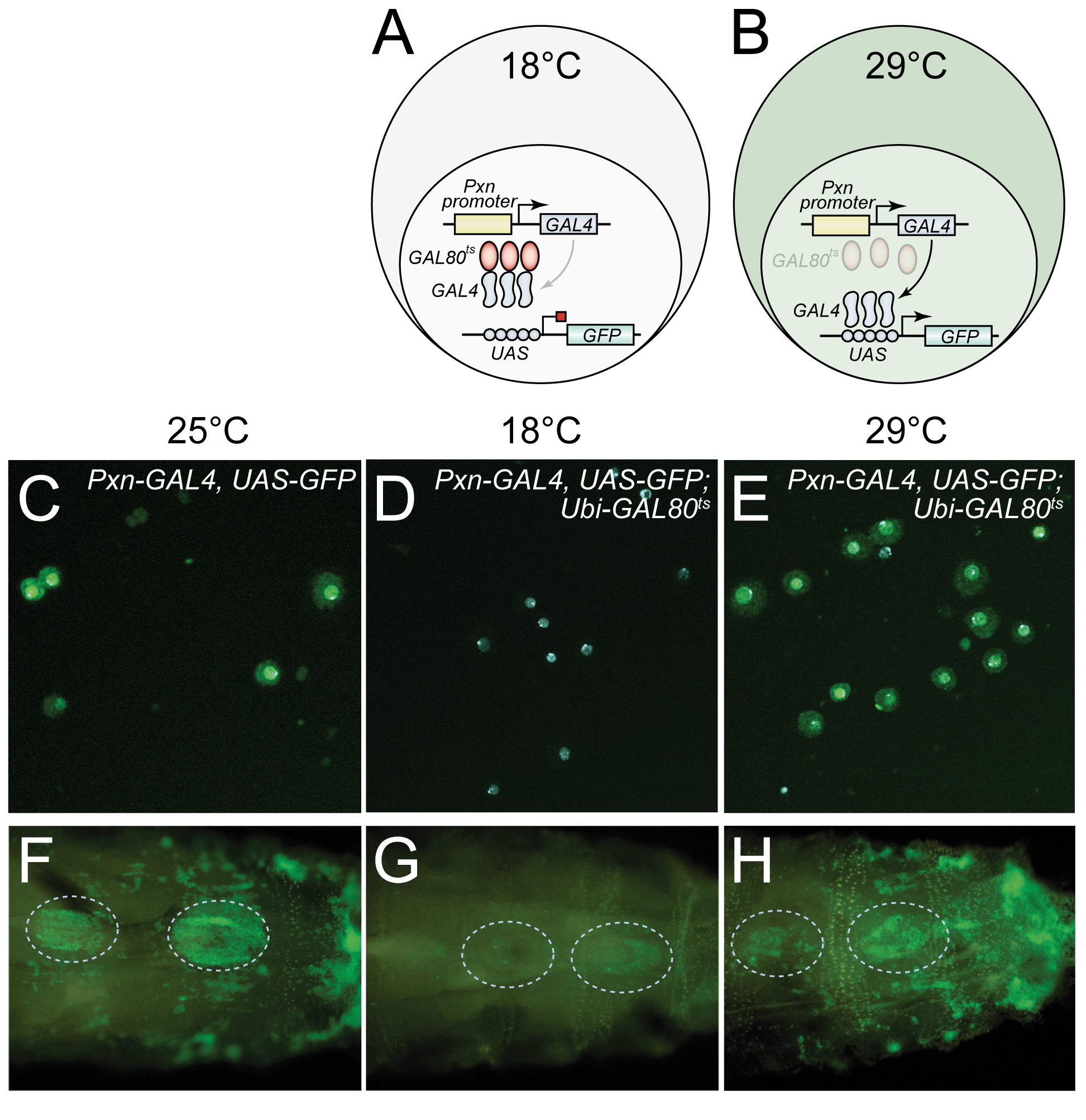

Supplement: Figure S3 — Control of Pxn-GAL4 mediated expression by GAL80ts. (A,B) Schematic illustrating the TARGET system. (A) A ubiquitously expressed, temperature-sensitive GAL80 variant (GAL80ts) is functional at 18°C. GAL80ts binds to and represses GAL4 expressed by Pxn-GAL4 in plasmatocytes, to repress GAL4-mediated transcription of responders like UAS-GFP. (B) At 29°C, GAL80ts is inactivated and GAL4 expressed by Pxn-GAL4 in plasmatocytes can activate transcription of targets like UAS-GFP. (C-H) (GAL80ts) can regulate Pxn-GAL4 mediated gene expression in hemocytes. Hemocytes were isolated from (C) Pxn-GAL4, UAS-GFP third instar larvae, (D) Pxn-GAL4, UAS-GFP; GAL80ts third instar larvae raised 18°C, and (E) Pxn-GAL4, UAS-GFP; GAL80ts third instar larvae raised at 18°C but shifted to 29°C for 24 hr. Hemocytes were stained with anti-GFP antibodies (green) and DAPI (blue) to reveal nuclei. GFP is detected in Pxn-GAL4, UAS-GFP hemocytes, and Pxn-GAL4, UAS-GFP; GAL80ts hemocytes at 29°C, but is not detected in hemocytes from Pxn-GAL4, UAS-GFP; GAL80ts larvae raised at 18°C. GFP expression in (C) and (E) is equivalent. (F–H) Live intact larvae of the equivalent genotypes to (C–E) showing GFP fluorescence in dorsal sessile hemocyte compartments (dashed ellipse). (F) GFP-expressing hemocytes are detected in sessile compartments in Pxn-GAL4, UAS-GFP larvae. (G) The sessile patches are indistinguishable from the surrounding tissues at 18°C but (H) become clearly visible after 24 hr temperature upshift at 29°C. (4.40 MB TIF) [file pone.0014051.s003.tif]
