# Supplementary material for: Lineage Tracing of Lamellocytes Demonstrates Drosophila Macrophage Plasticity
Source: PLoS One. 2010 Nov 19;5(11):e14051. doi: 10.1371/journal.pone.0014051 (PMC2988793; doi:10.1371/journal.pone.0014051)
Supplement: Table S1 — Primers used for real-time PCR analysis. (0.10 MB DOC) [file pone.0014051.s004.doc]

**Table S1**

**Primers used for real-time PCR analysis**

| ***Gene*** | ***Primer name*** | ***Primer sequence (5’ to 3’)*** | ***Product length (bp)*** |
| --- | --- | --- | --- |
| *ProPo-A1* | *ProPo-A1-5P* | GATACTCGCGCGCTACAATG | 124 |
|  | *ProPo-A1-3p* | GGTTATTCGTGCTGGACAGG |  |
| *Crq* | *Crq-5P* | gcgatcatcgaagcgggaag | 107 |
|  | *Crq-3P* | gcattagcttctgatggctc |  |
| *Pxn* | *Pxn-5P* | ctatcgctcgaatcgctagT | 100 |
|  | *Pxn-3P* | ggttatcaccaagtgtggtc |  |
| *Hml* | *Hml-5P* | ccgatgatgacgacgaggat | 100 |
|  | *Hml-3P* | gatgttgaagctaatgtggc |  |
| *Filamin-240* | *Fil-5P* | CGGATCAGTACGAGGAGAAC | 131 |
|  | *Fil-3P* | GATCGATGGTCTTCAGGTGC |  |
| *Ush* | *Ush-5P* | GAGTGCTCAGGATATAGGCG | 125 |
|  | *Ush-3P* | GCATCTTCATCAGCGTCGTC |  |
| *a-PS4* | *aPS4-5P* | ACACCGACTCCTTGACCATC | 128 |
|  | *aPS4-3P* | TGAGCACGTTGGTTAGCTTG |  |
| *a-PS5* | *aPS5-5P* | ACTTCGGTTACTCCGTGGTG | 106 |
|  | *aPS5-3P* | GCACCCACGTCATAGGAATC |  |

| *Mys* | *Mys-5P* | GATCACGGTACATGCGAGTG | 131 |
| --- | --- | --- | --- |
|  | *Mys-3P* | GTACCATGACCGGAGCAGAT |  |
| *β-Int-ν* | *B-Int-5P* | CTCGCCGGCAACTACTTAAC | 120 |
|  | *B-Int-3P* | GGACAGCCTGATCACTGGTT |  |
| *gcm* | *gcm-5P* | GGAGGAAGACGTGTGCGAAG | 142 |
|  | *gcm-3P* | GAAGCACCCATCCTTCTGCG |  |
| *lz* | *lz-5P* | ctccaactccatcagcatct | 137 |
|  | *lz-3P* | CCAATCCGAGTCCGAGTCCG |  |
| *lectin-24A* | *lectin24-5p* | CAATGCCTACAGCCAGGATT | 120 |
|  | *lectin24 -3P* | AGGCTAGGTGACCTCCCATT |  |
| *zfh1* | *zfh1-5P* | CCCAACCAGCCAGGTGCCGCTGCAGATGCC | 571 |
|  | *zfh1-3P* | AGTTGCGGCGGCAGCAGTTGCTGCATGGCC |  |
| *TepI* | *TepI-5P* | ctgaagtctcagtcagcctgactggacctt | 119 |
|  | *TepI-3P* | CGTAATCGCCTTCTGTTAGCTTCGGAATGT |  |
| *CG42640* | *CG42640-5P* | AGAGCGTGGCGGTGTACGCCAGGGATCGCG | 499 |
|  | *CG42640-3p* | CTTGGGGAAGTAGCCCTCGGCAATTGGTTC |  |
| *TepIV* | *TepIV-5P* | GTCAATGTCCATCTGGACTC | 143 |
|  | *TepIV-3P* | GAAGTCCTTGAGATCCATGG |  |

| *eater* | *eater-5P* | CGTCTGTCAATGCCTGACGG | 142 |
| --- | --- | --- | --- |
|  | *eater-3P* | AGACACCTTCCAGCTTCGTG |  |
| *chn* | *chn-5P* | TTACTCCTCGGAGCAGCAGT | 214 |
|  | *chn-3P* | CCATCTTGTTCTCGCCGACT |  |
